# Learning curve of autonomous computer-assisted implant surgery systems for postgraduate dental trainees: an in vitro study

**DOI:** 10.1186/s12903-025-07569-9

**Published:** 2026-01-05

**Authors:** Zixin Luo, Tianru Xu, Ying Yang, Jiangyong Huang, Zhe Wu, Ping Li

**Affiliations:** 1 School and Hospital of Stomatology, Guangdong Engineering Research Center of Oral Restoration and Reconstruction, Guangzhou Medical University, Guangzhou, 510182 China; 2 Guangzhou Key Laboratory of Basic and Applied Research of Oral Regenerative Medicine, Guangzhou Medical University, Guangzhou, 510182 China

**Keywords:** Robotic computer-assisted implant surgery, Learning curve, Accuracy, Time, Digital dentistry

## Abstract

**Objectives:**

This study investigated the accuracy and learning curve of dental postgraduate trainees in robot-assisted implant surgery (r-CAIS) and examined the characteristics of learning curve.

**Materials and methods:**

After receiving comprehensive training, eleven dental trainees used r-CAIS to place three implants, 3 trials per day over four experimental days, with each experimental day occurring every 72 h. Trueness was assessed by comparing postoperative model scans with preoperative planning. The total time was recorded during the experiment and subsequently categorized into preparation and surgery times.

**Results:**

The average deviations for platform, apex, and angular were 0.91 ± 0.39 mm, 0.93 ± 0.38 mm, and 1.38 ± 0.61°, respectively. The mean total time, preparation time, and surgery time were 954.36 ± 222.69 s, 321.36 ± 119.30 s, and 632.53 ± 140.30 s, respectively. Analysis indicated that there were no statistically significant differences in trueness between different experimental days and trials for platform, apex, and angular (p-values: Day: 0.0924, 0.2735, 0.3223; Trial: 0.6647, 0.7873, 0.5270). However, all time measures (total, preparation, and surgery time) showed significant reductions as days and trials increased (p-values < 0.0001 for all). Additionally, there was a significant interaction between days and trials for surgery time (*p* = 0.0123). Normality tests revealed that the trueness data and surgery time were not normally distributed on day 1, but conformed to a normal distribution on the rest days.

**Conclusion:**

The results demonstrated stability in trueness during the learning phase of r-CAIS, although there could still be a learning curve in terms of time metrics. Additionally, the accumulation of experience may exert varying influences during the preparation and surgical phases.

**Clinical significance:**

The r-CAIS can enable dental trainees to achieve high trueness during their initial exposure to surgical procedures. Understanding the differing learning processes associated with various r-CAIS procedures can help develop more effective training programs and promote the broader clinical applications of r-CAIS.

## Introduction

Advancements in computer-assisted implant surgery (CAIS) have promoted increasingly widespread clinical applications by simplifying implant procedures and enabling personalized, predictable, and efficient treatments while reducing complications [1–3]. Static (s-CAIS) and dynamic (d-CAIS) computer-assisted implant surgeries represent the first and second generations, respectively, with robotic (r-CAIS) representing the latest advancement [4]. Several previous studies demonstrated that r-CAIS achieved higher trueness than freehand, s-CAIS, and d-CAIS approaches, with this trueness being particularly pronounced in single-tooth implant placements, and even in complex scenarios such as immediate implant placement and edentulous jaws [5–9]. Trueness was closely associated with reduced complications and protection of critical anatomical structures, both essential for successful restorations [10, 11]. Therefore, r-CAIS showed promising clinical potential. As r-CAIS continues to evolve, understanding the associated learning curve becomes increasingly significant to maximize its effectiveness in clinical practice.

The concept of the learning curve is fundamental in evaluating surgical proficiency since it represents the rate of skill acquisition and improvement through repeated practice [12]. In digital implant technology, it typically reflects how increasing the number of training procedures enhances the trueness and efficiency of implant placement. This is especially important for r-CAIS since it is still in its early stages of clinical application with limited adoption thus far [13]. Surgeons’ proficiency is critical for achieving optimal clinical outcomes using a robotic-assisted laparoscopic surgery system [14]. Therefore, understanding the learning curve associated with r-CAIS is essential for adopting training strategies, especially for dental trainees, to minimize potential clinical risks and facilitate broader implementation of this emerging technology [15, 16].

Previous studies into the learning curves of s-CAIS and d-CAIS have provided important insights, indicating significant improvements in d-CAIS in both operating time and implant trueness over successive procedures, often reaching a plateau after a series of cases [17–19]. In contrast, s-CAIS exhibited less pronounced improvements in these aspects [17, 20]. Despite these findings, existing studies are limited by small sample sizes and a predominance of in vitro data, necessitating further research. Compared to s-CAIS and d-CAIS, investigations into the learning curve of r-CAIS are scarce. Two recent studies on the effects of training frequency and practitioner experience on robotic implant trueness reported consistently high trueness levels [21, 22]. Nevertheless, detailed learning curve patterns and associated challenges in clinical settings have not been fully elucidated.

This study investigated the impact of increased training repetitions on implant trueness and surgical duration in robotic implant placement to construct a detailed learning curve. Given the close association between learning curves and operator training, the study specifically focused on dental trainees in clinical dentistry to better understand the education and proficiency development required for r-CAIS. Therefore, the first null hypothesis was that the training days and each trial do not affect the trueness of r-CAIS. The second null hypothesis was that neither the training days nor each trial impacts the operational time of r-CAIS.

## Materials and methods

### Preparation of dental models and designation of the implant’s position

**Fig. 1:**
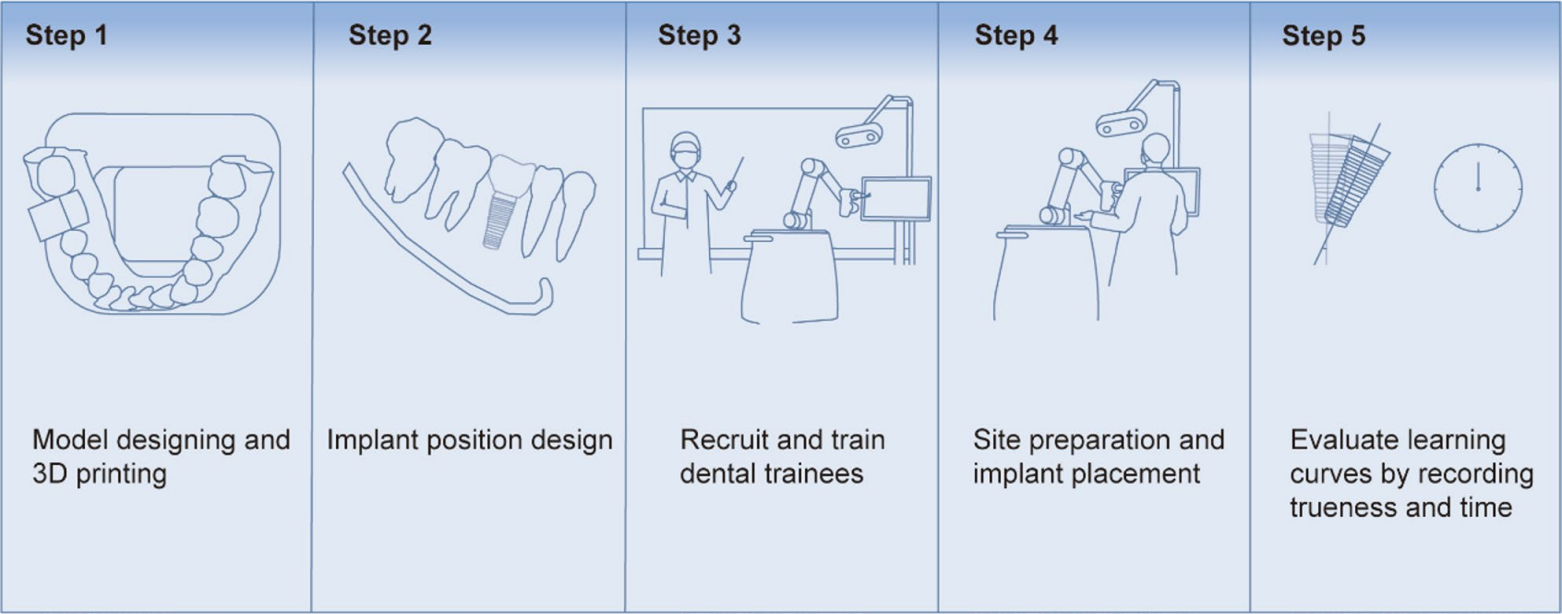
Flowchart of the experimental procedure

**Fig. 2:**
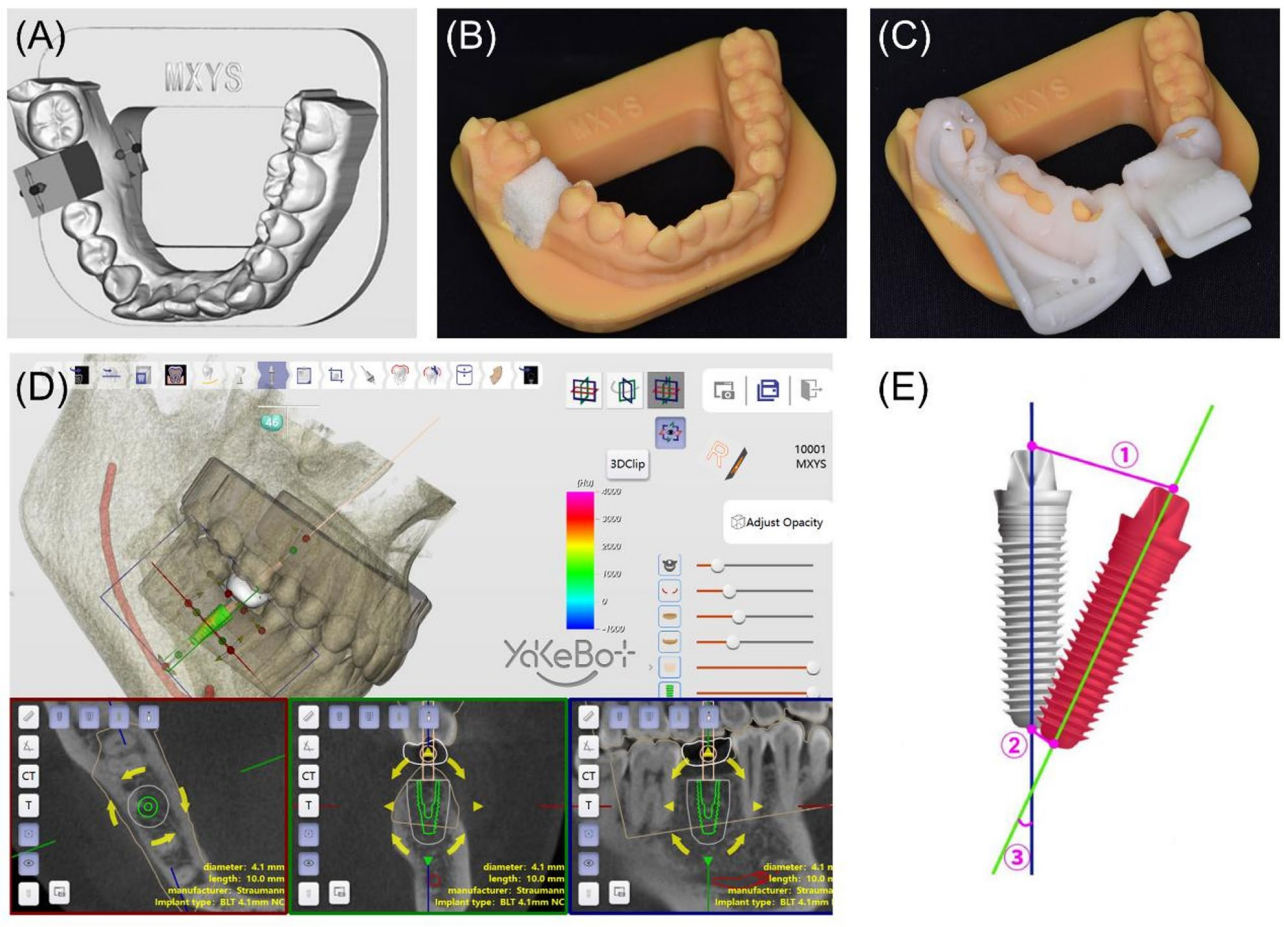
Pre-experimental preparation. **A** Schematic representation of the dental model with the position of tooth #46 evaluated. **B** A uniform resin block was placed into the implant site. **C** The guide plate was installed and confirmed to be fully seated. **D** Design of the implant position. **E** Graphical representation of the measured discrepancies between the planned and placed implants. Trueness parameters: ① angular deviation; ② global platform deviation; ③ global apex deviation

Fig. 1 illustrates the experimental workflow. Cone-beam computed tomography (CBCT) was used to capture the patient’s dental arch and export the data in DICOM (digital imaging and communications in medicine) format. Additionally, an intraoral scanner 3Shape Trios (3Shape A/S, Copenhagen, Denmark) obtained scans of the maxillary and mandibular arches in STL (Stereolithography) format. Both DICOM and STL files were subsequently imported into the DentalNavi software version 1.1.2.1456 (Beijing Yakebot Technology Co., Ltd., Beijing, China) for alignment. Within the software, tooth #46 was digitally removed to simulate single-tooth loss. An experienced dentist with over 30 years of implant experience collaborated with a software engineer to conduct virtual implant planning, including implant type and drilling sequences (Fig. 2D).

Further modifications were made to the dental model in the software: (1) A base compatible with the simulation head model was added to the mandibular dental arch model to facilitate securing the dental arch model onto the head model. (2) A rectangular cavity measuring 2.0 × 1.0 × 1.0 cm was hollowed out at the alveolar ridge position corresponding to tooth #46, intended for accommodating the resin block for implant. Five identical mandibular dental arch models were printed using photosensitive resin (Jianghe, China) through 3D printing (PioNext D158, Chuangxiang 3D, China). Polyurethane foam blocks (simulating type-2 of the maxilla and mandibular bone) were purchased and laser-cut into small rectangular blocks measuring 2 × 1 × 1 cm in dimension. These resin blocks fit precisely into the hollowed-out sections of the mandibular dental arch models, providing excellent mechanical stability [23] (Figs. 2A-C).

### Recruitment and training of participants

The total sample size was determined based on previous in vitro studies of r-CAIS learning curves using PASS software (version 15), which indicated that 40 implants were required with an alpha of 0.05 and a power of 0.9 [21]. To enhance the rigor of the experiment, 11 postgraduate dental trainees, each with at least two years of clinical experience and a valid license, were recruited based on prior research on dynamic navigation, resulting in a total of 121 implants used [17]. Trainees were formally recruited and they voluntarily agreed to participate after being fully informed about the study’s content. Despite proficiency in basic dental procedures, the trainees had no prior experience in implant surgery. Each participant performed one experimental procedure every 72 h (three days), with a total of four procedure days, during which three implants were placed per day [24]. The procedure days were categorized as Days 1, 2, 3, and 4, with each day’s experimental procedures were divided into three trials: the first, the second, and the third.

Before the first implant procedure, volunteers received theoretical and practical training in both freehand and r-CAIS. An experienced implant dentist conducted freehand training, while robot-assisted training was led by engineers and instructors. Minimal prior training was provided to thoroughly investigate the complete learning curve of r-CAIS and the challenges faced by dental trainees. Following theoretical instruction and on-site demonstrations, the volunteers practiced hands-on with both techniques and successfully placed an implant before undertaking the official trials.

### Implant placement and operating time recording

The dental implant robotic system used in this study (Yakebot Technology Co., Ltd, Beijing, China) comprises a robotic arm, an infrared vision system, and a real-time dynamic navigation screen [25]. The system can autonomously perform entry into and exit from the oral cavity, site preparation, and implant placement. At the same time, the operator can change drill bits, select commands, and monitor the screen.

**Fig. 3:**
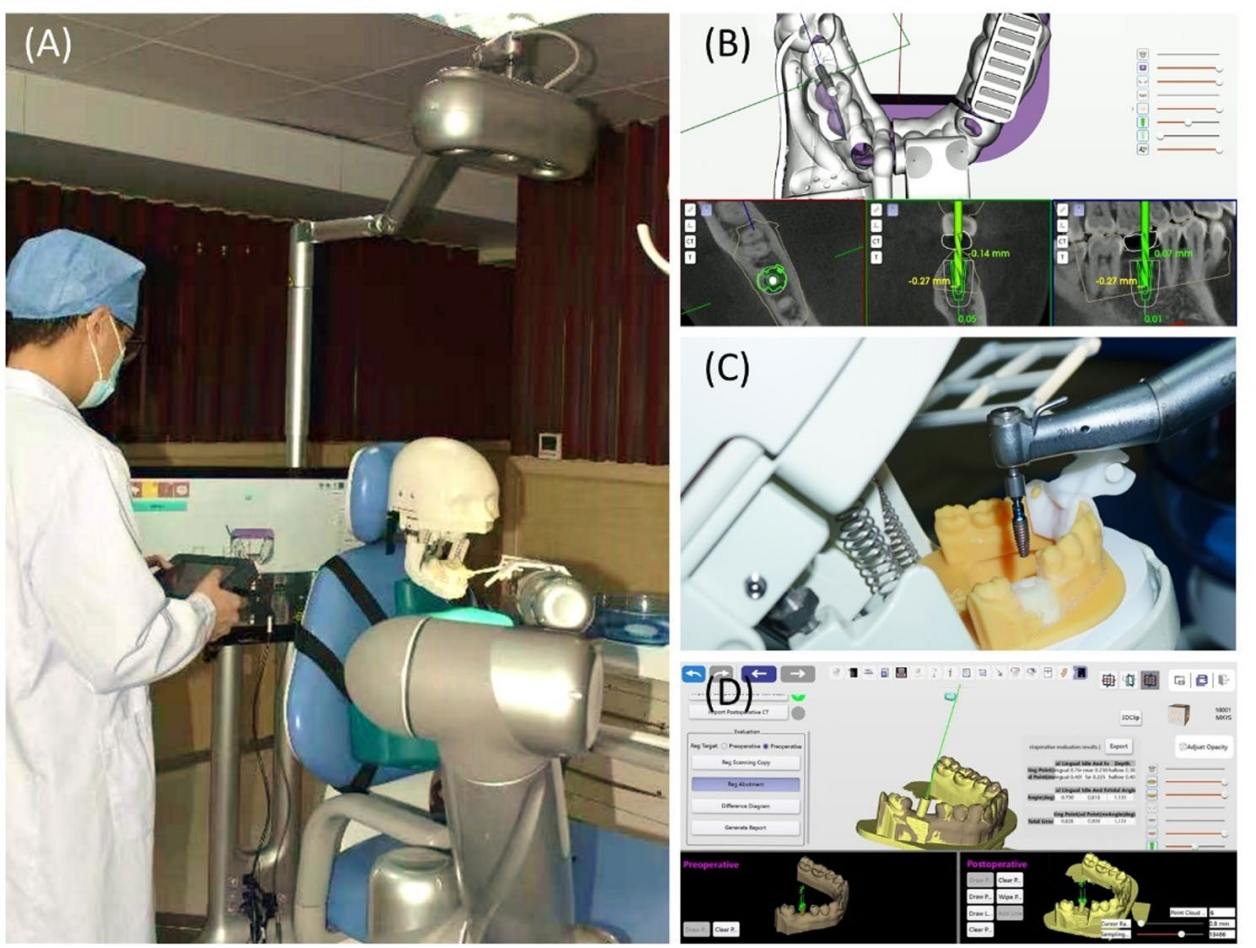
Experimental procedure. **A** A specialty trainee performing dental implant surgery using the robotic system. **B** Dynamic tracking of the drill bit position as displayed on the intraoperative screen. **C** The robotic arm autonomously placed the implant. **D** Matching postoperative intraoral scan files with preoperative planning

The formal experimental procedure was as follows [5]:

1. Preoperative preparation: The experimenters secured the simulation head model on the dental chair and adjusted the infrared vision system, robotic arm, and dynamic navigation screen to appropriate positions (Fig. 3A). The dental arch model was then fixed within the simulation head model.

(2) Registration: The volunteers operated the control screen as the surgery commenced. Using the infrared vision system, the robot automatically identified the positions of the robotic arm and the implant handpiece to complete the registration process. The registration of the disk guide was manually performed by the volunteers using a serpentine probe (calibration probe).
(3) Calibration and pathway input: The volunteers fixed the positioning marker on the dental arch model and ensured it was in position. The positioning marker consisted of two parts: one around the missing tooth area with guide points, and the other around the lower incisors with guide points and a groove for connection with the positioning marker. The volunteers used the serpentine probe’s tip to sequentially contact the registration pits on the positioning marker to complete the calibration process. By establishing connectivity between the robotic arm, dental model, vision system, and CBCT images, the screen could reveal jawbone anatomy, implant path, and drill positions in real-time. Subsequently, volunteers manipulated the robotic arm to record the entry pathway.
(4) Implant process: The robotic arm was automatically moved and drilled per the preset protocol. The volunteers only needed to select the steps and manually change the corresponding drills. After the drilling, the implant was manually loaded onto the handpiece and automatically placed by the robotic arm (Figs. 3B-C).
(5) Recording the operating time: The experimenters recorded the operation time (from the beginning of registration to the end of implant placement) and categorized it into preparation time (from the beginning of registration to the end of pathway input) and implant time (from the end of pathway input to the end of implant placement).

After each implant placement, the resin block containing the implant was removed, labeled, and replaced with a new resin block in the dental model for the next implant.

### Postoperative intraoral scanning and evaluation

The resin block with the implant was placed into the dental model, and a scan was performed using the scanner. The postoperative STL file was imported into the DentalNavi. The postoperative image was superimposed on the preoperative image by selecting three identical key points on both of them. The trueness results were automatically evaluated by the software, encompassing platform, apex, and angular deviations based on the implants’ central axis [26]. The distance deviations were measured in mm, and the angular deviation was measured in degrees (Fig. 2E). Two proficient experimenters skilled in software operation independently matched the preoperative and postoperative STL files to minimize matching errors. The final assessment was based on the average of the evaluations from these two individuals, and the intraclass correlation coefficient (ICC) was calculated for the assessment results of the two evaluators.

### Statistical analysis

Statistical analyses were performed using GraphPad Prism 9 (GraphPad Inc., USA) with the significance threshold set at *p* < 0.05. Trueness and time were treated as continuous variables and reported using means and standard deviations, followed by being subjected to normality testing using the Shapiro-Wilk test. The interaction between experimental days and trial counts was initially assessed using interaction plots. A two-way analysis of variance (ANOVA) was conducted to evaluate the main effects of experimental days and trial counts on trueness and time. In case of significant ANOVA results, post hoc Tukey tests were applied for multiple comparisons. Finally, Spearman’s rho correlation coefficient was calculated to explore the relationships between experimental days, trial counts, trueness, and time.

## Results

### Trueness

**Fig. 4:**
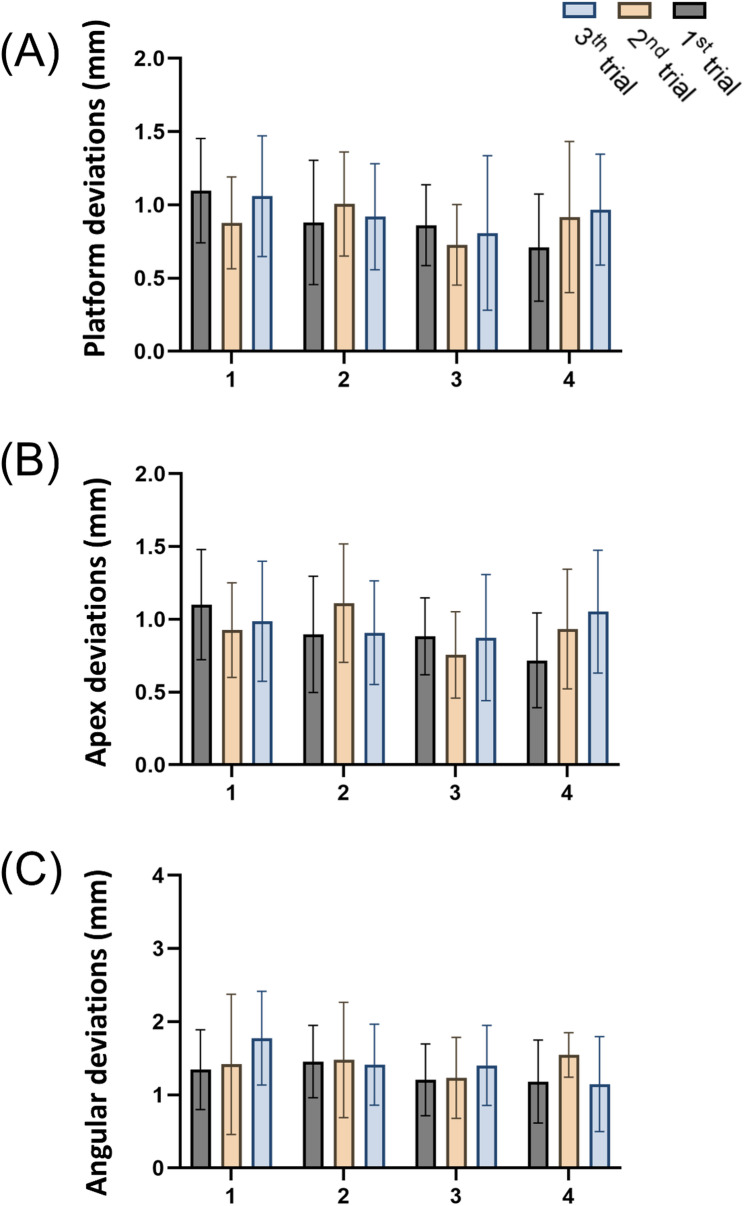
Mean implant trueness across trials. The implant trueness was evaluated for eleven dental trainees over four days, with three trials each day. The bar chart presents (**A**) platform deviation, (**B**) apex deviation, and (**C**) angular deviation

The means and standard deviations for overall platform, apex, and angulation deviations were0.91 ± 0.39 mm, 0.93 ± 0.38 mm, and 1.38 ± 0.61°, respectively. The analysis of the ICC between the two examiners showed that the inter-observer measurements of the 3D deviations and angular deviations of the implant platform and apex were 0.909, 0.833, and 0.728, respectively, indicating good consistency. The Shapiro-Wilk test was conducted on the trueness grouped by days, indicating that the overall platform, apex, and angular deviations did not follow a normal distribution on day 1 (*p* < 0.05), while they complied with normal distribution on days 2, 3, and 4 (*p* > 0.05) (Table 1). A two-way ANOVA was conducted to assess the influence of experimental days and trials on trueness, which revealed no significant interaction between experimental days and trials for any type of deviation (platform deviations: F (6, 120) = 0.9986, *p* = 0.4296; apex deviations: F (6, 120) = 1.365, *p* = 0.2342; angular deviations: F (6, 120) = 0.8994, *p* = 0.4979). Additionally, no significant differences were observed in any deviation types between different experimental days and trial counts (*p* > 0.05), suggesting that r-CAIS is not significantly affected by variations in training days and trial counts. Furthermore, none of the deviation types demonstrated any correlation with changes in days and trials (*p* > 0.05) (Table 2). The average platform, apex, and angular deviations across different days and trials were displayed using bar charts (Fig. 4).

**Table 1:** Trueness and time outcomes by day (mean ± SD), with per-Day Shapiro–Wilk normality test results. (* indicates *p* < 0.05.)

	Day	1	2	3	4
		Attempt (Day) (means ± SD)			
Trueness					
Platform deviation (mm)		1.02 ± 0.36	0.95 ± 0.39	0.78 ± 0.36	0.88 ± 0.43
Apex deviation (mm)		1.00 ± 0.37	0.97 ± 0.39	0.84 ± 0.33	0.91 ± 0.40
Angular deviation (°)		1.51 ± 0.74	1.45 ± 0.61	1.28 ± 0.52	1.29 ± 0.54
Time (s)					
Preparation time		403.1 ± 155.4	330.4 ± 111.9	284.8 ± 71.06	267.2 ± 71.77
Surgery time		752.4 ± 158.3	654.7 ± 117.0	571.4 ± 93.58	549.8 ± 81.36
Total time		1155 ± 267.1	985.1 ± 180.9	857.0 ± 113.4	816.9 ± 115.3
Normality by Day (Shapiro–Wilk p, summary)					
Trueness					
Platform deviation		0.0233*	0.3812	0.5637	0.4378
Apex deviation		0.0284*	0.7594	0.5357	0.6953
Angular deviation		0.0498*	0.4276	0.8967	0.3308
Time					
Preparation time		0.6588	0.0906	0.4160	0.4463
Surgery time		0.0297*	0.2449	0.3617	0.2566
Total time		0.3602	0.4976	0.1947	0.3815

**Table 2:** Inferential results for trueness and time outcomes: two-way ANOVA (day, trial, and day × trial) and spearman associations with day and trial. (* indicates *p* < 0.05.)

Outcome	Interaction: day × trial (*p*)	Main effect: day (*p*)	Main effect: trial (p)	Association with day (Spearman ρ, *p*)	Association with Trial (Spearman ρ, *p*)
Trueness					
Platform deviation	0.4296	0.0924	0.6647	0.0644	0.4884
Apex deviation	0.2342	0.2735	0.7873	0.1447	0.5280
Angular deviation	0.4979	0.3223	0.5270	0.0838	0.3094
Time					
Preparation time	0.2200	< 0.0001*	< 0.0001*	< 0.0001*	< 0.0001*
Surgery time	0.0123*	< 0.0001*	< 0.0001*	< 0.0001*	< 0.0001*
Total time	0.0035*	< 0.0001*	< 0.0001*	< 0.0001*	< 0.0001*

### Time

The average total, preparation, and surgery times for the implants were 954.36 ± 222.69 s, 321.36 ± 119.30 s, and 632.53 ± 140.30 s, respectively. The Shapiro-Wilk test was conducted on the time grouped by days, indicating that surgery time was not normally distributed on day 1 (*p* < 0.05) but was normally distributed on days 2, 3, and 4 (*p* > 0.05). This may indicate that the impact of individual differences on surgery time diminished as the days increased. Both preparation time and total time were normally distributed across all days (*p* > 0.05) (Table 1). In the two-way ANOVA test, the interaction between days and trials was significant for both surgery time and total time (surgery time: F (6, 120) = 2.860, *p* = 0.0123; total time: F (6, 120) = 3.452, *p* = 0.0035), while no interaction was found for preparation time (F (6, 120) = 1.400, *p* = 0.2200) (Table 2). This may suggest that preparation time was independently influenced by days and trials, while the accumulation of experience from increasing trials affects surgical time on subsequent days.

Analyzing day and trial as main effects revealed significant differences in preparation time, surgery time, and total time. Correlation analysis further demonstrated significant associations between preparation time, surgery time, and total time with both day and trial (Day: preparation time *p* < 0.0001, *r* = -0.4266; surgery time *p* < 0.0001, *r* = -0.5540; total time *p* < 0.0001, *r* = -0.5776; Trial: preparation time *p* < 0.0001, *r* = -0.4157; surgery time *p* < 0.0001, *r* = -0.3773; total time *p* < 0.0001, *r* = -0.4612), indicating that all time parameters decrease with the increase in experimental days and trials (Table 2).

**Fig. 5:**
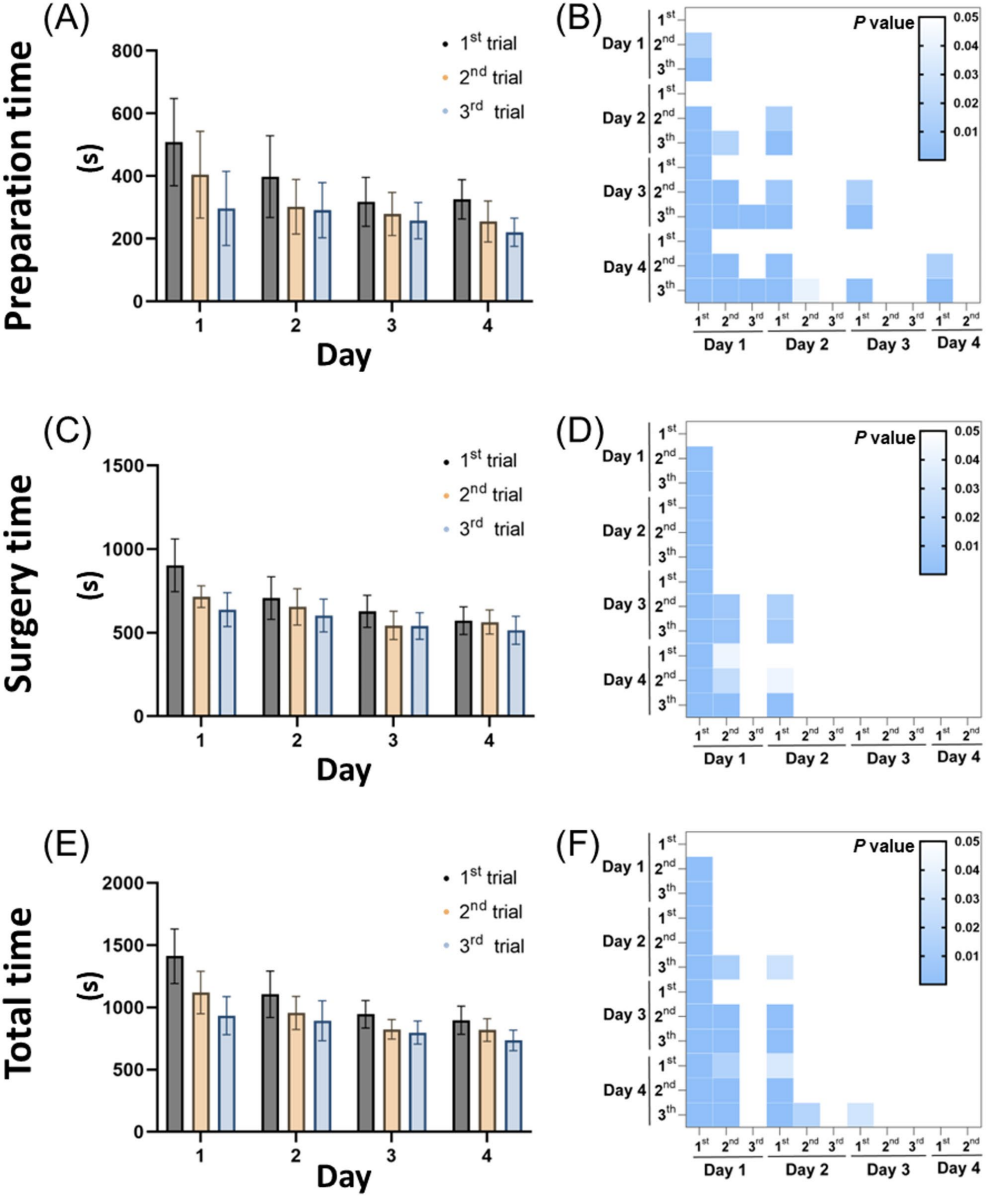
Average time per trial and statistical differences. Times for implants performed by eleven dental trainees over four days, with three trials each day, were recorded. The bar chart shows (**A**) preparation time, (**C**) surgery time, and (**E**) total time. The results showing statistically significant differences between the experiments are presented as heat maps for (**B**) preparation time, (**D**) surgical time, and (**F**) total time. Blue boxes indicate statistically significant differences (*p* < 0.05), and white boxes indicate non-significant differences (*p* > 0.05)

The average time results under different days and trials were displayed using bar charts, illustrating a gradual decrease with an increase in daily practice sessions (Figs. 5A, C, and E). However, a slight increase was observed during the first trial on the following day, which might be attributed to skill decay resulting from the interval between sessions. Comparisons of time results across different days and trials were illustrated using statistical heat maps (Figs. 5B, D, and F). Significant differences in preparation time were observed on each day, while significant differences in surgery time and total time predominantly appeared on days 1 and 2. Considering the influence of interactions, this phenomenon may suggest that the consistent impact of trials across different days led to uniform and sustained improvement in preparation time. In contrast, the interaction between days and trials in surgery time indicated a potential effect of experience accumulation, characterized by rapid improvement during the early stages and slower growth in later stages.

## Discussion

This study investigated the learning curve associated with r-CAIS for dental trainees. No significant changes in trueness were observed as the number of experimental days and trials increased across four experimental days, during which three implants were placed daily. Therefore, the first hypothesis was accepted. Conversely, preparation time, surgery time, and total time decreased as the number of experimental days and trials increased, refuting the second hypothesis.

In this study, trueness did not show significant changes for dental trainees as they practiced more. This could be attributed to the fully autonomous robotic system since it minimizes human factors, particularly by precisely controlling the robotic arm and providing real-time visual feedback. These features facilitated fine adjustments and effectively mitigated hand tremors during implant preparation and placement [27]. Consequently, r-CAIS reduced the influence of the operator’s skill level, diminishing the adverse effects on trueness typically observed during the initial learning phase. Similarly, previous research involving novice clinicians using r-CAIS also reported no significant differences between different trials [3, 28]. In contrast, studies involving d-CAIS demonstrated marked improvements in trueness as practice increased [19, 29]. It may be inferred that the trueness of robotic systems is less influenced by the operator’s skill level [30, 31]. however, further studies are needed to confirm this observation. Moreover, in the normality test of the trueness assessments, day 1 did not conform to a normal distribution, while days 2, 3, and 4 did, possibly because repeated training weakened the impact of individual differences.

Regarding temporal metrics, significant differences in preparation, surgery, and total times were observed across varying experimental days and trials. Overall, the recorded average total time was comparable to that in previous r-CAIS clinical studies, with the average total time for the latter trials being shorter [32], which might be attributed to the simplified nature of the model experiments compared to real clinical surgeries [28]. Previous studies on r-CAIS have also demonstrated an association between increased training repetitions and decreased total time [21]. Although the robotic system exhibits stability and high trueness, the operator’s proficiency in the procedure of r-CAIS continues to affect time metrics, indicating that a learning process still exists concerning the operational steps [2]. This study divided the total operative time into preparation time and surgery time for separate evaluation to further investigate the learning patterns associated with different operative phases of r-CAIS.

In the preparation time, the data from days 1 to 4 conformed to a normal distribution, indicating minimal influence of individual differences on operations. However, due to the small sample size and limited trials, further research with a larger cohort is needed to investigate the impact of individual differences on the learning curve associated with r-CAIS. The two-way ANOVA showed no interaction between days and trials for preparation time, suggesting that increasing trials during this phase did not significantly affect subsequent days. This may be attributed to the standardized procedures for the registration and calibration of the robotic arm and the patient’s dental arch, largely governed by the machine’s programming [33]. Consequently, the influence of accumulated trainee experience appeared minimal. This trend was reflected in the statistical heat maps (Fig. 5), where significant differences in preparation time were consistently across days 1 to 4, particularly during the first trial of each day, potentially influenced by the forgetting curve effect, indicating similar trial count effects across all days [24]. It can be inferred that the standardization in the preparation phase reduced the impact of accumulated experience, resulting in a slow yet stable decline in the learning curve for preparation time. The limited sample size and study duration did not demonstrate a clear learning plateau in preparation time, suggesting that larger controlled studies are necessary to validate these findings.

In the analysis of surgery time, day 1 did not conform to a normal distribution, whereas days 2 to 4 did, indicating that individual differences were more pronounced during the initial learning phase and diminished as the study progressed. The two-way ANOVA indicated an interaction between days and trials, implying that the accumulation of trials influenced subsequent days. Significant differences in the statistical heat map were concentrated between days 1 and 2, with no significant differences observed in later days. This pattern reflected a rapid decline early on, followed by a slower decline, contrasting with the consistent decrease seen in preparation time. The closer relationship between surgery procedures and specialized implant knowledge may explain the greater influence of accumulated experience [33]. The lack of statistical differences in surgery time during the last two days could indicate a learning plateau. However, given the complexity of surgical knowledge and the potential impact of multiple factors (e.g., CBCT variations, calibration errors, and positioning plate displacements), this plateau may also represent a bottleneck period [34]. Further studies with extended duration and larger sample sizes are required to confirm the learning curve patterns in the surgical phase.

Only one previous study on d-CAIS explored the learning curves for preparation and surgery times, indicating that surgical experience could shorten the learning process for the d-CAIS calibration phase. However, it did not differentiate between the learning processes associated with preparation and surgery times [5]. Significant differences between the operative workflows of r-CAIS and d-CAIS necessitate further comparative research to elucidate the specific learning patterns across different phases of r-CAIS.

The literature on digital implantology reveals several key points regarding the learning curves of different technologies. Regarding s-CAIS, although placement trueness improves with practice, it does not exhibit a typical learning curve pattern [20]. The learning curve of d-CAIS is influenced by both the operator’s experience and the system’s automation level, with active systems reaching proficiency faster than passive ones [29, 35]. The r-CAIS is associated with improved procedural efficiency over time; however, its trueness remains stable [21, 22]. These findings suggest that future research should differentiate between learning effects related totrueness and procedural efficiency and more closely examine the interaction between the operator’s experience and system automation. Additionally, expanding research in real clinical settings with larger sample sizes will further clarify learning patterns with digital implant technologies.

Currently, research on the learning curves of clinical surgical robots has become relatively systematic, covering a wide range of procedures such as visceral and soft tissue procedures represented by the Da Vinci system, minimally invasive vascular anastomosis, and orthopedic applications like knee arthroplasty [25]. Compared with dental implant robots, both the above systems exhibit certain similarities in learning curve characteristics, including rapid early improvements followed by a plateau phase, and a decisive influence of factors such as technical attributes, user interface design, and the trueness of three-dimensional imaging and navigation systems on learning outcomes [36]. However, key differences also exist: clinical robots are applicable to a broader spectrum of procedures, they entail more complex workflows, and they require more multidisciplinary team collaboration [37]. Their learning curve assessments also emphasize multidimensional, proficiency-based criteria; in contrast, dental implant robots are primarily used in single-operator settings and are mainly evaluated based on trueness and efficiency [38]. Furthermore, simulation-based training and stepwise competency assessment systems have been widely adopted in the clinical robotic field, while these approaches remain to be further developed for dental implant robots [39, 40].

This study had some limitations. First, the sample size of operators was relatively small, and the duration of the experiment day was short, possibly limiting the understanding of the learning curve for r-CAIS. Additionally, the trueness observed was lower than the values reported in previous studies [7], and there were some individual data points with significant deviations, possibly due to the limited surgical experience of the dental trainees. Furthermore, the in vitro experiments were unable to fully replicate the clinical surgical scenarios, suggesting that the learning patterns of r-CAIS may differ in actual clinical settings. Further research is necessary to compare the learning curves between different digital technologies and investigate potential factors influencing the learning curve for r-CAIS, such as surgical experience, the autonomy level of the robotic system, and training methods.

## Conclusions

This study investigated the learning curve and factors influencing dental trainees’ use of r-CAIS. In terms of trueness, no statistically significant changes were observed as practice increased, indicating that r-CAIS could maintain a stable, high trueness even in the early stages of learning. Regarding time efficiency, the total duration decreased with increasing days and trials. On Day 1, trueness and surgery time deviated from a normal distribution. On subsequent days, they confirmed, suggesting repetitive training reduced individual differences’ impact. Surgery time showed an interaction between days and trials, whereas preparation time did not, indicating accumulated experience affects surgery more. The learning curve for the preparation phase exhibited a continuous, regular change, while the surgical phase demonstrated a rapid decrease followed by a deceleration, highlighting the distinct characteristics between the two learning curves.

## Data Availability

The datasets used and/or analysed during the current study are available from the corresponding author on reasonable request.
